# Obesity-induces Organ and Tissue Specific Tight Junction Restructuring and Barrier Deregulation by Claudin Switching

**DOI:** 10.1038/s41598-017-04989-8

**Published:** 2017-07-11

**Authors:** Rizwan Ahmad, Bilal Rah, Dhundy Bastola, Punita Dhawan, Amar B. Singh

**Affiliations:** 1 Department of Biochemistry and Molecular Biology, University of Nebraska Medical Center, Omaha, NE 68198 USA; 2 Department of Bioinformatics, University of Nebraska Omaha, Omaha, USA; 3 VA Nebraska-Western Iowa Health Care System, Omaha, NE USA

## Abstract

Obesity increases susceptibility to multiple organ disorders, however, underlying mechanisms remain unclear. The subclinical inflammation assisted by obesity-induced gut permeability may underlie obesity-associated co-morbidities. Despite eminent clinical significance of the obesity led gut barrier abnormalities, its precise molecular regulation remains unclear. It is also unknown whether barrier deregulations, similar to the gut, characterize other vital organs in obese individuals. The claudin family of proteins is integral to the tight junction (TJ), the apical cell-cell adhesion and a key regulator of the epithelial barrier. Using comprehensive physiological and biochemical analysis of intestinal and renal tissues from high-fat diet fed mice, critical for maintaining metabolic homeostasis, this study demonstrates that profound TJ-restructuring by organ and tissue-specific claudin switching characterize obese organs. Protein expression and cellular distribution were examined. In-silico analysis further highlighted potential association of select claudins, modulated by the obesity, with signaling and metabolic pathways of pathological significance. *In vitro* studies using Leptin or DCA-treatment suggested causal significance of obesity-induced changes in tissue microenvironment in regulating barrier deregulations in tissue-specific manner. Overall, current findings advances our understanding of the molecular undertakings of obesity associated changes that help predispose to specific diseases and also identifies novel windows of preventive and/or therapeutic interventions.

## Introduction

Obesity is a serious metabolic disorder that predisposes individuals to and/or increases susceptibility to multiple pathological conditions including gastrointestinal disorders and cancers, diabetes and renal pathologies^1^. At current, 36.5% adults in the U.S.A. are obese and the annual medical cost of managing obesity was estimated to be a whopping $147 billion as far back as in 2008^2–4^. However, once considered a problem only in high-income countries, overweight and obesity is now dramatically on the rise in low- and middle-income countries, particularly in urban settings^5^. To prevent this social and economic catastrophe, we need immediate measures that also require improved molecular understanding of the problem. In this regard, a common trait identified in obese individuals and associated diseases is the prevalence of leaky/hyper-permeable gut^6^. Additionally, the heightened antigen/immune interaction due to leaky gut can impose local or systemic inflammatory burden in obese individuals to increase susceptibility to specific diseases. However, despite its apparent clinical significance, molecular undertaking of increased mucosal leakiness in obese individuals remain poorly understood. It is also not clear whether obesity associated barrier deregulation is limited to the gut epithelium or it is systemic. Importantly, barrier deregulation is central to undesired antigen-immune interaction and inflammation.

Tight junction (TJ), the principal determinant of epithelial paracellular permeability, helps to regulate epithelial barrier properties^7^. While it is organized by specific interactions between a wide spectrum of structural, adapter and signaling proteins, the integral role of claudin family of transmembrane proteins in regulating tight junction’s structure/function is now well-established^8^. Importantly, not all claudin proteins are alike and they differ drastically in their ability to regulate trans-epithelial resistance, a measure of an epithelial monolayer integrity and/or paracellular ion transport^9^. For instance, claudin-1 expression in epithelial cells increases TER while claudin-2 expression decreases TER and increases paracellular permeability for ions and non-charged small molecules^10^. In accordance, in mammalian body, claudin-2 is expressed only among leaky epithelia including the intestinal crypts and proximal tubular epithelium, and is specifically absent from tighter epithelia like collecting duct and urinary bladder^11, 12^. Moreover, studies from our as well as other laboratories have demonstrated that permeability enhancing disease conditions markedly increase claudin-2 expression^13–15^. However, detailed analysis of the modulation of claudin proteins in gut epithelia and other organs in correlation with obesity induced gut hyper-permeability is lacking.

In the current study, we have performed comprehensive physiological and biochemical *in vitro* and *in vivo* analyses to demonstrate that obesity induced by the intake of high fat diet induces gut hyper-permeability by tissue-specific claudin switching in obese gut epithelium compared to lean subjects. We further demonstrate that obesity induced claudin switching and tight junction restructuring is organ specific and may depend on macro-environmental changes. We anticipate the outcome to improve molecular understanding of obesity associated disorders, and help improve clinical management of the problem.

## Results

### High fat diet induces obese phenotype and glucose intolerance

**Figure 1:**
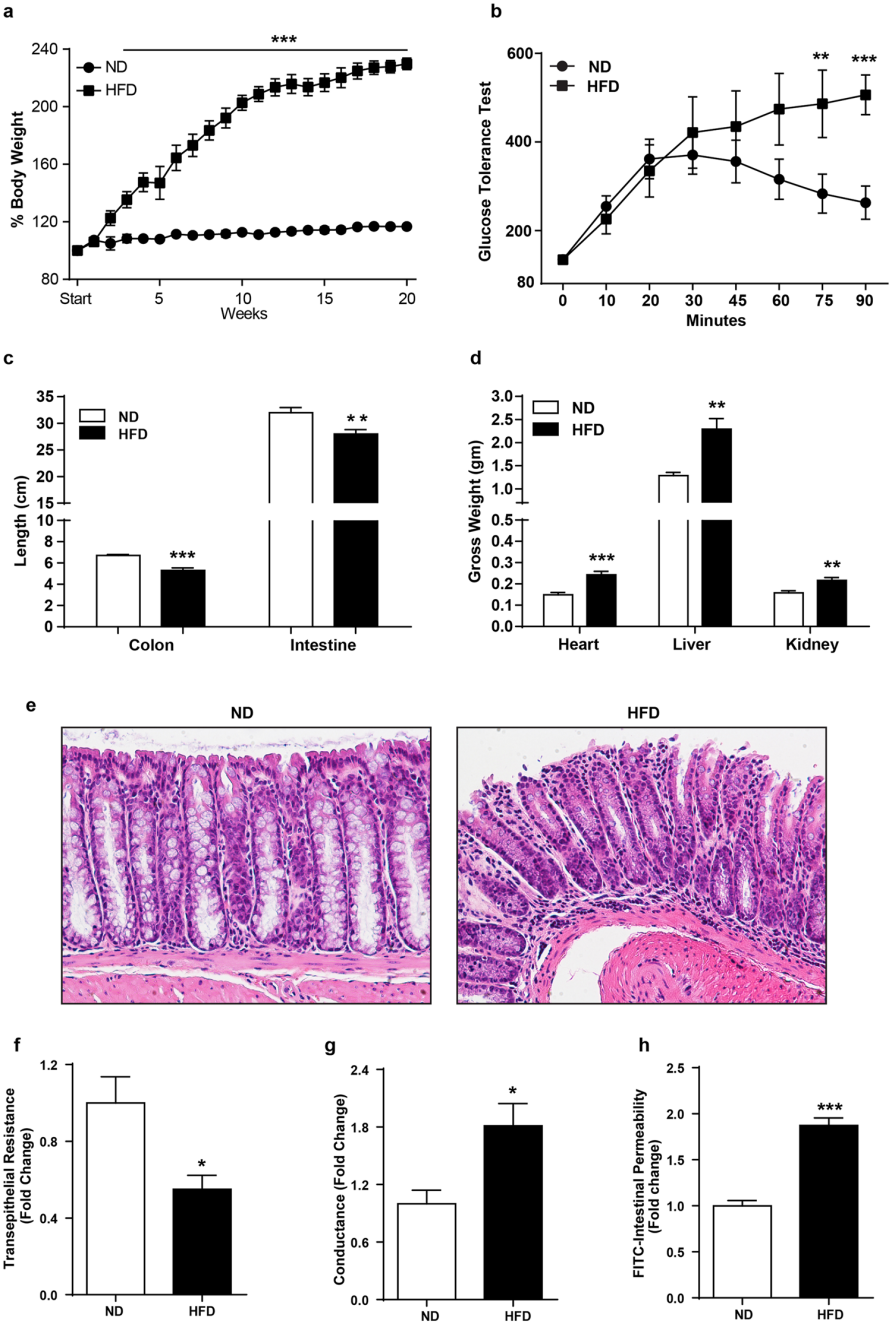
High fat diet induces obesity and associated metabolic and physiological changes in C57/BL6 mice. *8*–*10 weeks old mice were subjected to normal chow or high fat diet for 20 weeks* (*N* = *8*). (**a**) body weight changes in mice over the period of ND- or HFD-diet feeding; (**b**) Oral glucose tolerance test (OGTT); (**c)** Changes in colonic and intestinal length in HFD-versus ND-fed mice; (**d**) Changes in organ weight in HFD-versus ND-fed mice; (**e**) Representative images showing immune cell infiltration in mice colon with HFD- versus ND-mice. (**f** and **g**) Colonic trans-epithelial resistance and conductance across the mucosal sheet changes during HFD-mice versus control; (**h**) Intestinal permeability (for FITC-dextran) in HFD versus ND-mice. Values are presented as mean ± SEM ***P ≤ 0.001, **P ≤ 0.01 and *P ≤ 0.05 compared to control mice on ND.

Mice included in the study were similar in age (8–10 weeks old; N = 8), body weight, and male/female ratio, between the study groups. As per expectation, body weight gain in HFD-fed mice was appreciably higher after as little as 2 weeks of feeding (versus ND-mice; p < 0.05; Fig. 1(a)), which became significantly higher 6 weeks post-feeding of HFD diet (versus ND-mice; p < 0.001) (Fig. 1(a)). Remarkably, when sacrificed at 20 weeks post-HFD feeding, obese mice also demonstrated significant increases in liver (p < 0.01), heart (p < 0.001) and kidney (p < 0.01) weight compared to the ND-fed mice (Fig. 1(d)). The oral glucose tolerance test (OGTT), the benchmark assay to determine insulin intolerance, conducted at week 20 post-HFD feeding, before sacrificing the mice, further demonstrated failure of the blood glucose clearance from blood (Fig. 1(b), p < 0.001)) in HFD-fed mice, suggesting chronic glucose intolerance. Taken together, these data confirmed validity of the high-fat diet feeding in inducing obesity and associated metabolic impediments.

### HFD-diet induces hyper-permeable gut and mucosal inflammation

We further examined if mice fed on high fat diet possessed leakier gut than ND-fed mice. The ND- and HFD-mice (after 18 weeks of feeding differential diet) were subjected to intestinal permeability assay, as described in “Materials and Methods”^13^. A significant increase (p < 0.001) in FITC-dextran levels, administered orally, in blood plasma in HFD-mice compared to ND-mice confirmed presence of leaky gut in HFD-mice (Fig. 1(h)). By using the Ussing chamber system mounted with freshly isolated mucosal epithelium, we further confirmed this finding which demonstrated a significant decrease in the TER in HFD-mice (p < 0.05) while conductance across the epithelium increased (p < 0.05) compared to the ND-mice (Fig. 1(f and g)). A leaky gut promotes mucosal inflammation^16^. Moreover, obesity induced inflammatory reprogramming is believed to promote susceptibility of obese individuals to metabolic disorders^17–19^. Therefore, we examined inflammatory signatures in HFD-fed mice gut versus ND-fed mice. Histological evaluation of the H&E slides of swiss-rolled intestine and colon indeed demonstrated areas of immune infiltration and epithelial damage in HFD-mice compared to the ND-mice (Fig. 1(e)). Significant decreases in the colon (p < 0.01) and small intestine (p < 0.001) lengths in obese mice (versus lean mice) further supported mucosal inflammation in HFD-mice compared to the ND-mice (Fig. 1(c). Taken together, our data demonstrated that high-fat diet induced obesity causes gut hyper-permeability and subclinical mucosal inflammation.

### Obesity induces claudin switching for tight junction restructuring in intestinal epithelium

**Figure 2:**
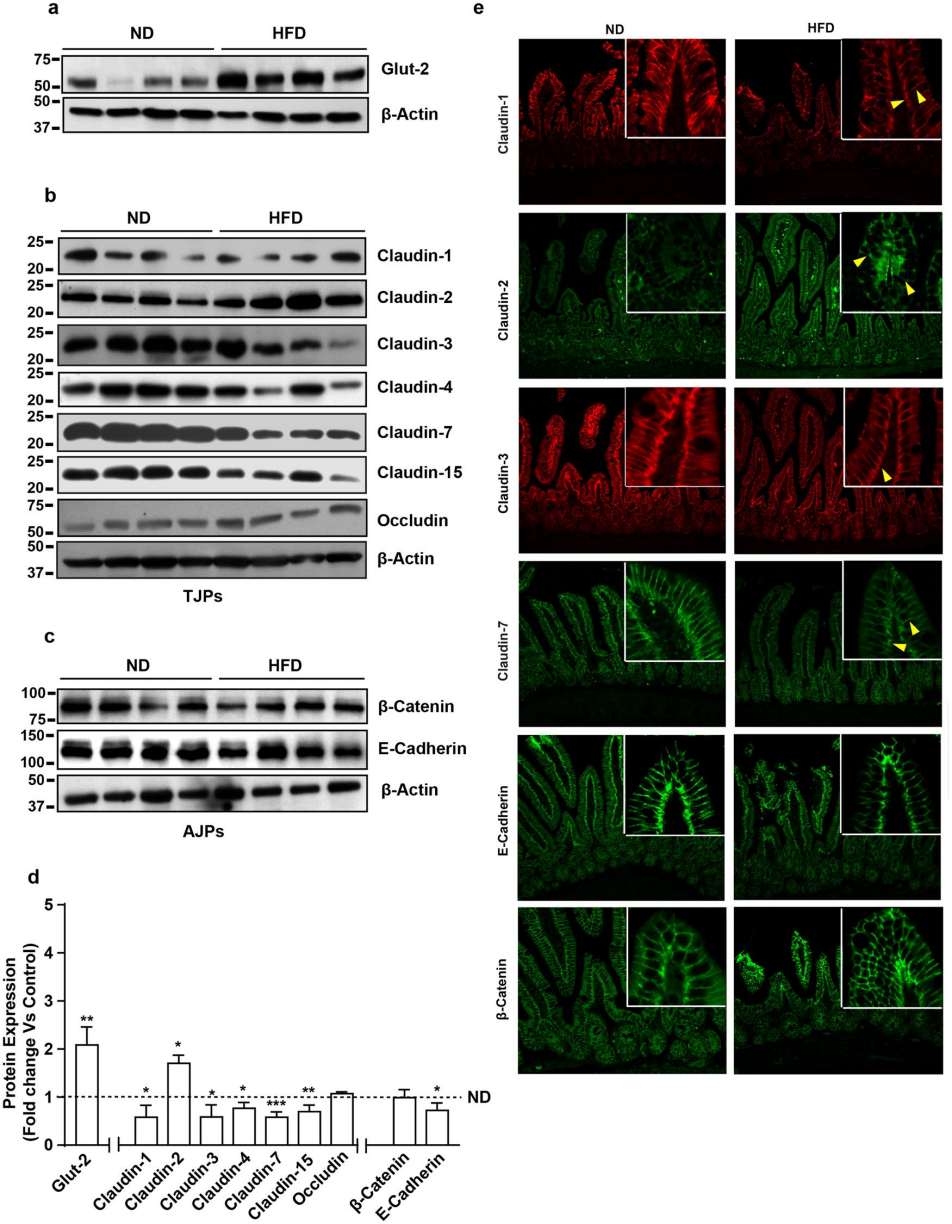
High fat diet induces restructuring of tight junction (but not adherent junction) to modulate mucosal barrier function in small intestine: *Immunoblot analysis of total tissue lysate prepared using small intestine from mice fed on normal chow or high fat diet for 20 weeks* (N = 4). (**a**) Glut-2 expression in HFD and ND-fed mice, as positive control; (**b**) Immunoblot analysis to determine changes in TJPs antigen-specific antibodies; (**c**) Immunoblot analysis to determine potential changes in AJPs (E-Cadherin and β-Catenin) in obese versus lean mice; (**d**) Quantitative analysis of immunoblot band intensity for respective protein; (**e**) Representative confocal immunofluorescent images of TJ and AJPs of small intestine from HFD- and ND-fed mice. Values are presented as mean ± SEM *P ≤ 0.05 compared to control (normal chow fed mice; ND) mice.

Mucosal barrier is a complex structure consisting of multiple epithelial and immune components. However, key role of the tight junction in this barrier regulation is widely recognized^13, 20–22^. Therefore, we performed a comprehensive analysis of tight junction integral proteins ﻿(TJPs). Concurrent analysis of adherent junction proteins (AJPs), E-cadherin and β-catenin, between HFD- and ND-mice helped determine specificity of the outcome. Glut-2 expression in HFD-mice versus ND-mice served as positive control which, as predicted, was significantly increased in HFD-mice intestine (versus ND mice, p < 0.001) (Fig. 2(a)). Immunoblot analysis demonstrated significant however diverse changes in claudin expression in HFD-mice versus ND-mice. In specific, expression of claudin-1 (p < 0.05), claudin-3 (p < 0.05), claudin-4 (p < 0.05), claudin-7 (p < 0.001) and claudin-15 (p < 0.01) decreased significantly in HFD-mice compared to the ND-mice (Fig. 2(b and d)). In contrast, expression of claudin-2, the leaky claudin protein, was significantly upregulated (p < 0.05) (Fig. 2(b and d)). In same samples, expression of occludin, yet another TJP or beta-catenin, an AJP, remained largely unaltered yet E-cadherin expression decreased significantly (verses ND-mice, p < 0.01) (Fig. 2(b, c and d)).

However, barrier function can be altered due to the changes in cellular content of barrier-associated proteins and/or their cellular distribution^3, 13, 23, 24^. Therefore, we further examined if expected membrane tethered cellular localization of proteins under investigation is also altered in obese mice intestine. Regrettably, immunostaining results for occludin and claudin-15 did not provide satisfactory outcome and were not pursued further. E-cadherin and β-catenin demonstrated expected lateral membrane localization in ND-mice intestine, which remained largely unaltered in HFD-mice intestine (Fig. 2(e)). Furthermore, in ND-mice claudin-1, -3 and -7 were expressed in luminal and apicolateral membrane localization (Fig. 2(e)). In HFD-mice, immunostaining for all three proteins was drastically downregulated however the loss in lateral membrane locations appeared robust than the luminal expressed protein. In contrast, claudin-2 immunoreactivity was minimal and predominantly luminal in the ND-mice, which increased sharply in HFD-mice and it was present in the luminal position and the cell cytoplasm (Fig. 2(e)). Taken together, our data suggested that obesity-induced gut leakiness associates with sweeping modulation of the barrier composition in small intestine epithelium.

### Obesity induced claudin switching is specific for the large intestine

**Figure 3:**
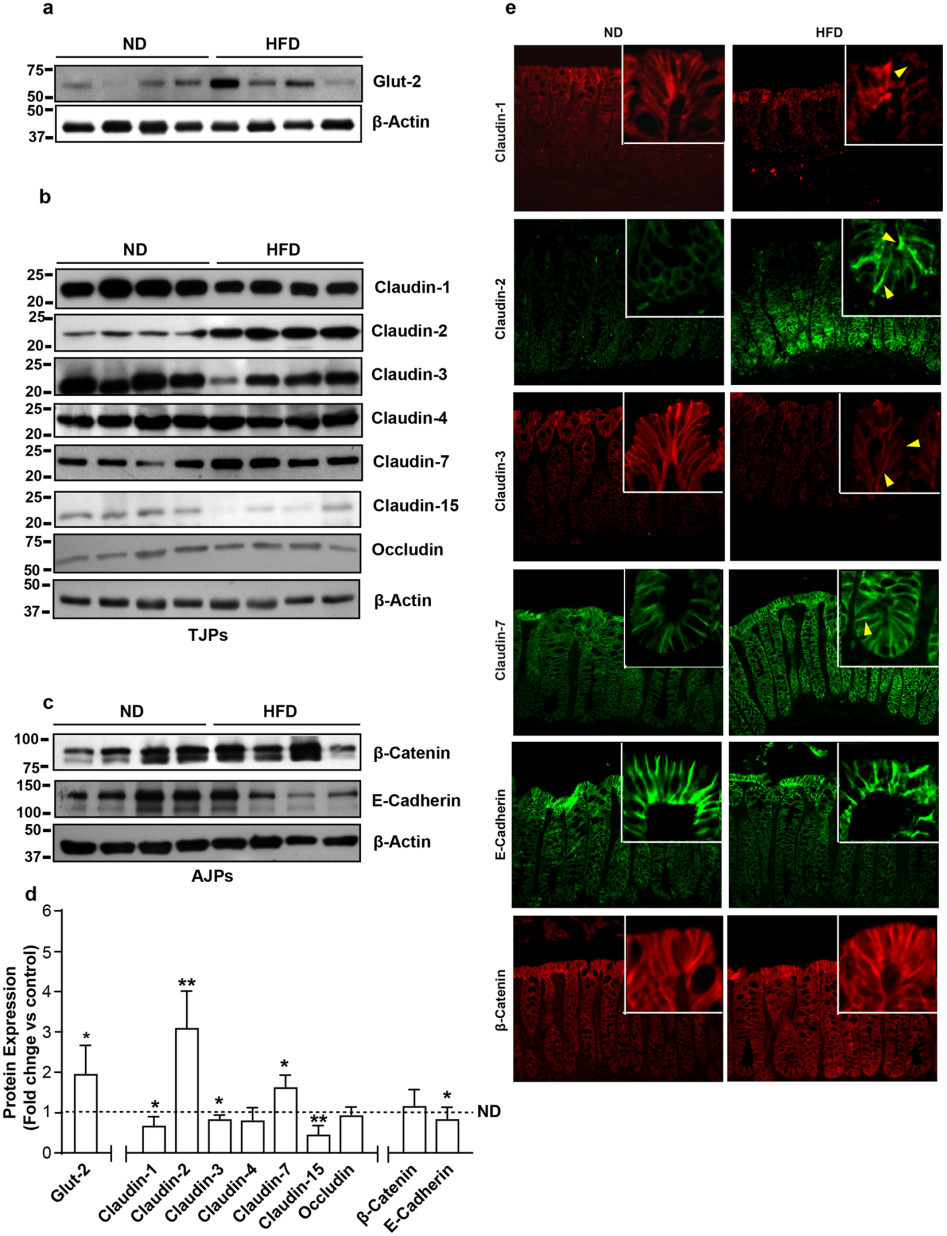
High fat diet induces colon specific restructuring of tight junction and adherent junction: *Immunoblot analysis of total tissue lysate prepared using the Colon from ND- and HFD-fed mice* (N = 4). (**a**) Glut-2 expression in HFD and ND-fed mice, as positive control; (**b**) Immunoblot analysis to determine changes in TJPs using antigen-specific antibodies; (**c**) Immunoblot analysis to determine changes in AJPs including E-cadherin and β-catenin; (**d**) Densitometry analysis of the immunoblot band intensity for respective protein; (**e**) Representative immunofluorescent images of TJ and AJPs of colon from HFD- and ND-fed mice. Values are presented as mean ± SEM **P ≤ 0.01; *P ≤ 0.05 compared to control (normal chow fed mice; ND) mice.

We further determined whether obesity induces similar changes in cell-cell adhesions in small and large intestine. Potential changes in above described proteins were determined in colonic epithelium. Immunoblot analysis using total colon lysate demonstrated significant upregulation of Glut-2 expression in HFD-mice (p < 0.05) compared to the ND-mice, as in small intestine (Fig. 3(a)). The changes in cell-cell adhesion proteins were also robust however differed sharply compared to the small intestine, suggesting tissue specificity. A significant decrease in claudin-1 (p < 0.05), claudin-3 (p < 0.05) and claudin-15 (p < 0.01) expressions in HFD-mice colon compared to the ND-mice was found (Fig. 3(b and d)). Claudin-4 and occludin expressions remained largely unaltered in these samples (Fig. 3(b and d)). However, contrasting the significant decrease in small intestine in HFD-mice, colonic claudin-7 expression was significantly upregulated in HFD-mice (versus ND-mice, p < 0.05). The pore forming claudin, claudin-2, was however significantly upregulated (versus ND-Mice, p < 0.01) in HFD-mice colon as in small intestine (Fig. 3(b and d)). Interestingly, the changes among AJPs in the colon were similar to the small intestine where E-cadherin levels in HFD-mice decreased significantly (p < 0.05) though cellular β-catenin content remained largely unaltered (Fig. 3(c and d)). Immunofluorescence microscopy revealed minimal claudin-2 expression at the base of the colonic crypt while claudin-3 expression was concentrated predominantly among differentiated colonocytes at the crypt top in ND-mice colon (Fig. 3(e)), as previously described^15^. The marked upregulation of claudin-2 expression in obese mice colon was also distributed to a higher crypt depth, and was expressed as both, membrane-tethered and cytosolic protein. In contrast, claudin-3 immunoreactivity in HFD-mice was simply suppressed (versus ND-mice). Both, claudin-1 and claudin-7 were present on the lateral membrane throughout the crypt however differed drastically in their intensity, with claudin-7 being the prominent protein Fig. 3(e)). In obese mice colon, despite an increase in intensity, claudin-7 expression remained localized predominantly at the lateral membrane. In contrast, the decrease in claudin-1 expression appeared more pronounced at lateral membrane locations compared to the luminal protein. Also, the lateral membrane localized E-cadherin and beta-catenin expressions in ND-mice colon appeared partially deregulated in HFD-mice colon. Taken together, our data demonstrated that obesity led restructuring of mucosal barrier is tissue specific and differs between the small and the large intestine epithelium.

### Obesity induced tight junction remodeling and claudin switching is organ specific

**Figure 4:**
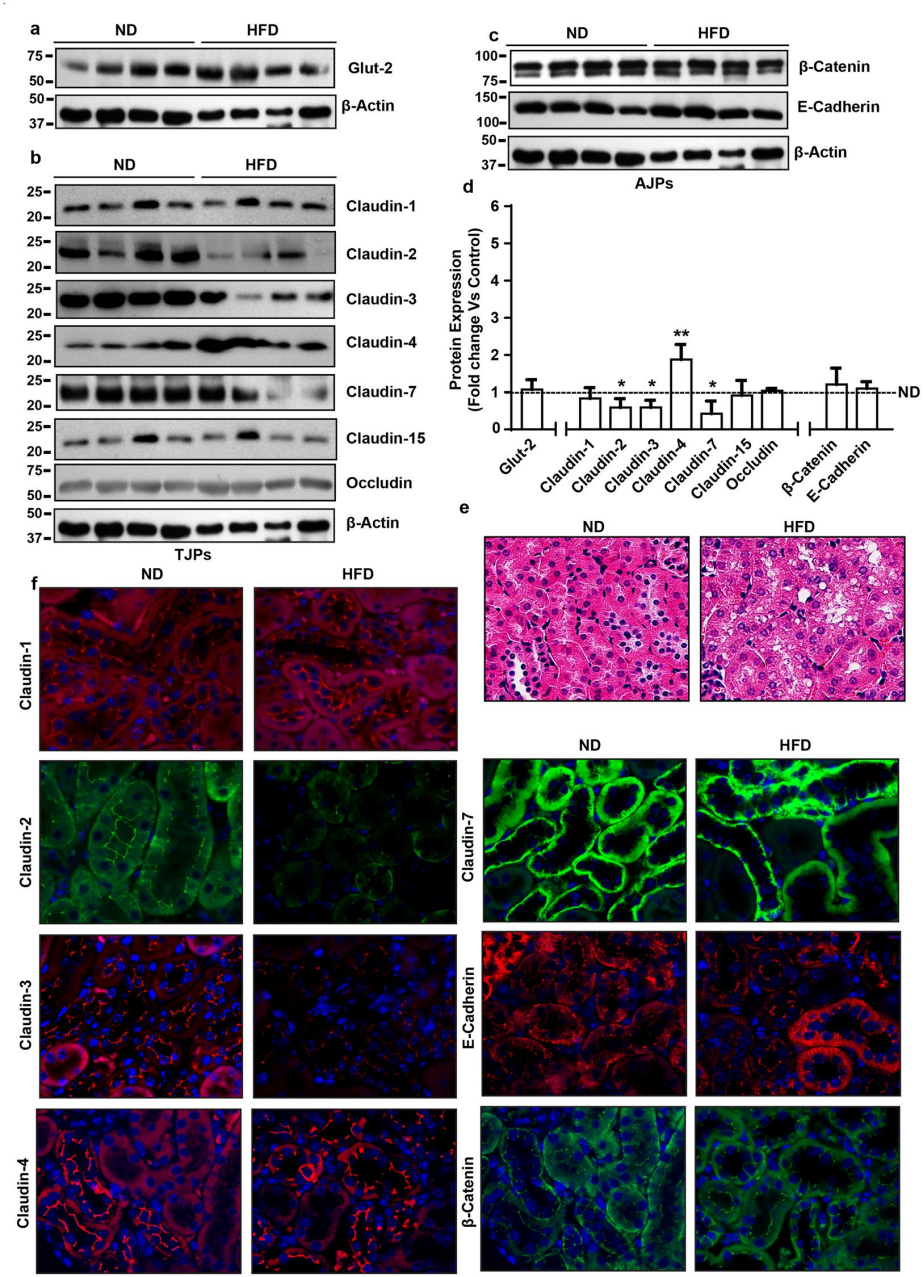
High fat diet induces contrasting (versus gut) changes in tight junction composition in renal epithelium: *Immunoblot analysis of total tissue lysate prepared using the kidney from ND- and HFD-fed mice* (N = 4). (**a**) Glut-2 expression in HFD and ND-fed mice, as positive control; (**b**) Immunoblot analysis to determine changes in TJPs using antigen-specific antibodies; (**c**) Immunoblot analysis to determine changes in AJPs including E-cadherin and β-catenin; (**d**) Quantitative analysis of immunoblot band intensity for respective protein; (**e**) Representative immunofluorescent images of specific TJPs using kidney sections from HFD- and ND-fed mice. Values are presented as mean ± SEM ***P ≤ 0.001; **P ≤ 0.01 and *P ≤ 0.05 compared to control (normal chow fed mice; ND) mice.

Obesity associated changes in the plasma glucose and triglyceride concentrations prime to hypertension and renal pathologies is evidenced by increased albuminuria, alterations in kidney morphology, and renal lipid accumulation^25^. Considering the newly defined role of claudin proteins in regulating paracellular ion homeostasis and the findings that genetic modification of claudin proteins lead to deregulated renal salt handling and functional impairment^26^, we also determined obesity-induced changes in claudin composition in the renal epithelium of HFD-mice versus ND-mice. As described, the kidney weight in HFD-mice increased significantly (versus ND-mice, p < 0.01) (Fig. 1(d)). Histopathological analysis of H&E slides further demonstrated signs of renal injury in HFD-mice (Fig. 4(e)). Immunoblot analysis using total kidney lysate further demonstrated robust changes in claudin proteins and tight junction composition in the renal epithelium, which however contrasted the changes in gut epithelium. No major changes were found specifically in claudin-1 expression in HFD-mice kidney versus ND-mice kidney (Fig. (b and d)). Similarly, claudin-15, a paracellular Na^+^ channel, expression and/or cellular distribution remained largely unaltered. However, expression of claudin-2 protein, also a paracellular Na^+^ channel expressed predominantly in the proximal tubular epithelium, decreased significantly in HFD-mice (versus ND-mice, p < 0.05) (Fig. 4(b and d)). Similar significant decreases in claudin-3 (p < 0.05) and claudin-7 (p < 0.05) expressions characterized renal epithelium in obese mice. In same samples, claudin-4 expression was markedly upregulated (versus ND mice, p < 0.01) (Fig. 4(b and d)). HFD-induced obesity did not affect occludin, E-cadherin or beta-catenin expressions to significant levels (Fig. 4(b and d)). Immuno-fluorescent analysis of candidate claudin proteins supported the outcome from immunoblot analysis (Fig. 4(f)). Taken together, our data demonstrated organ and tissue specificity for obesity-induced changes in claudin expression and tight junction restructuring.

### Deoxycholic acid (DCA) and leptin induce claudin switching to modulate barrier composition and apico-basal permeability in intestinal epithelial cells

**Figure 5:**
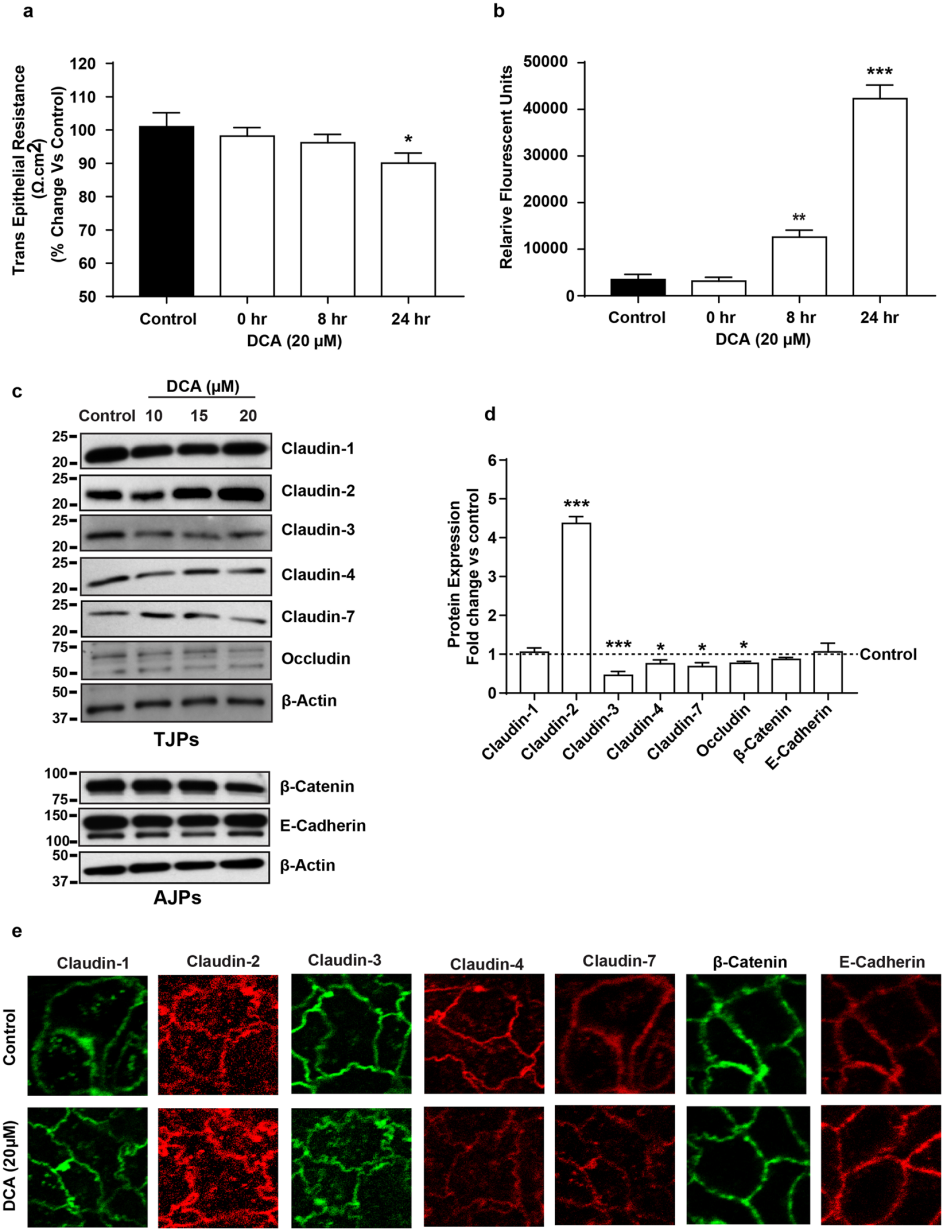
Exposure of intestinal epithelial cell (IEC) to Bile acid (DCA) modulates tight junction composition and barrier function similar to the HFD-induced tight junction restructuring in gut epithelium. *Polarized monolayer of Caco-2 cells was subjected to DCA (20 µM)-treatment (in complete culture medium) for different time-points*. (**a**) Effect of DCA-treatment upon TER across the cell monolayer; (**b**) Apico-basal paracellular permeability in control or DCA-treated cells as described in “materials and methods”. (**c**) Immunoblots analysis of total cell lysate from control and DCA treated cells by using antigen-specific antibodies; (**d**) Quantitative analysis of the antigen specific band intensity from immunoblot analysis; (**e**) Representative confocal immunoflurescent imaging to determine cellular localization of cell-adhesion proteins (TJPs and AJPs) in control and DCA-treated cells. Values are presented as mean ± SEM. ***P ≤ 0.001, **P ≤ 0.01 and *P ≤ 0.05 compared to control.

Dietary fat affects bile acid metabolism, excess fat processing and absorption requires increased amount of bile acids in the digestive tract. Accordingly, high-fat diet elevates bile acids concentration in the intestinal lumen. Remarkably, studies have further demonstrated direct association between orally delivered deoxycholic acid (secondary bile acid) and gut inflammation^27^. Similarly, leptin expression, secreted from the adipose tissue, is associated with obesity and obesity-associated mucosal inflammation^28, 29^. Therefore, in the light of our data from HFD-mice, we determined potential role/s of the gastrointestinal versus systemic secretomes in observed claudin switching and tight junction restructuring in obese mice. After optimizing the time (of treatment) and concentration of DCA that did not affect cell viability in polarized caco-2 cells (data not included), effects of DCA-treatment upon tight junction restructuring and function was determined. TER in cells cultured in the presence of DCA (20 µM) in culture medium decreased significantly (p < 0.05) (Fig. 5(a)). The DCA-treated cells also demonstrated a gradual increase in the apico-basal permeability, which became significantly higher at 8- and 24-hours post treatment data points (p < 0.001 versus control cells) (Fig. 5(b)). Further determinations using immunoblot analysis revealed a significant upregulation in claudin-2 expression (p < 0.001), as seen in obese mice gut epithelium while claudin-3 decreased markedly in DCA treated cells (p < 0.001) compared to the vehicle-treated cells (Fig. 5(c and d)). Similarly, significant decreases in the expression of other TJPs, claudin-4, claudin-7 and occludin (p < 0.05 for each proteins versus control cells) were also observed in DCA-treated cells (Fig. 5(c and d)). However, DCA-treatment did not affect claudin-1, occludin, beta-catenin and E-cadherin expressions (Fig. 5(c and d)). Immunofluorescence depicted intense claudin-2 staining at cell-cell junction along with vesicular cytosolic staining which further increased with DCA treatment. Membrane localized claudin-3, claudin-4 and claudin-7 proteins in control cells were largely decreased but it demonstrated cytosolic presence for claudin-3 protein (Fig. 5(e)). Collectively these results supported a critical role for increased bile acid secretion in response to increased dietary fat intake and, in promoting gut permeability by modulating claudins expression and tight junction restructuring.

**Figure 6:**
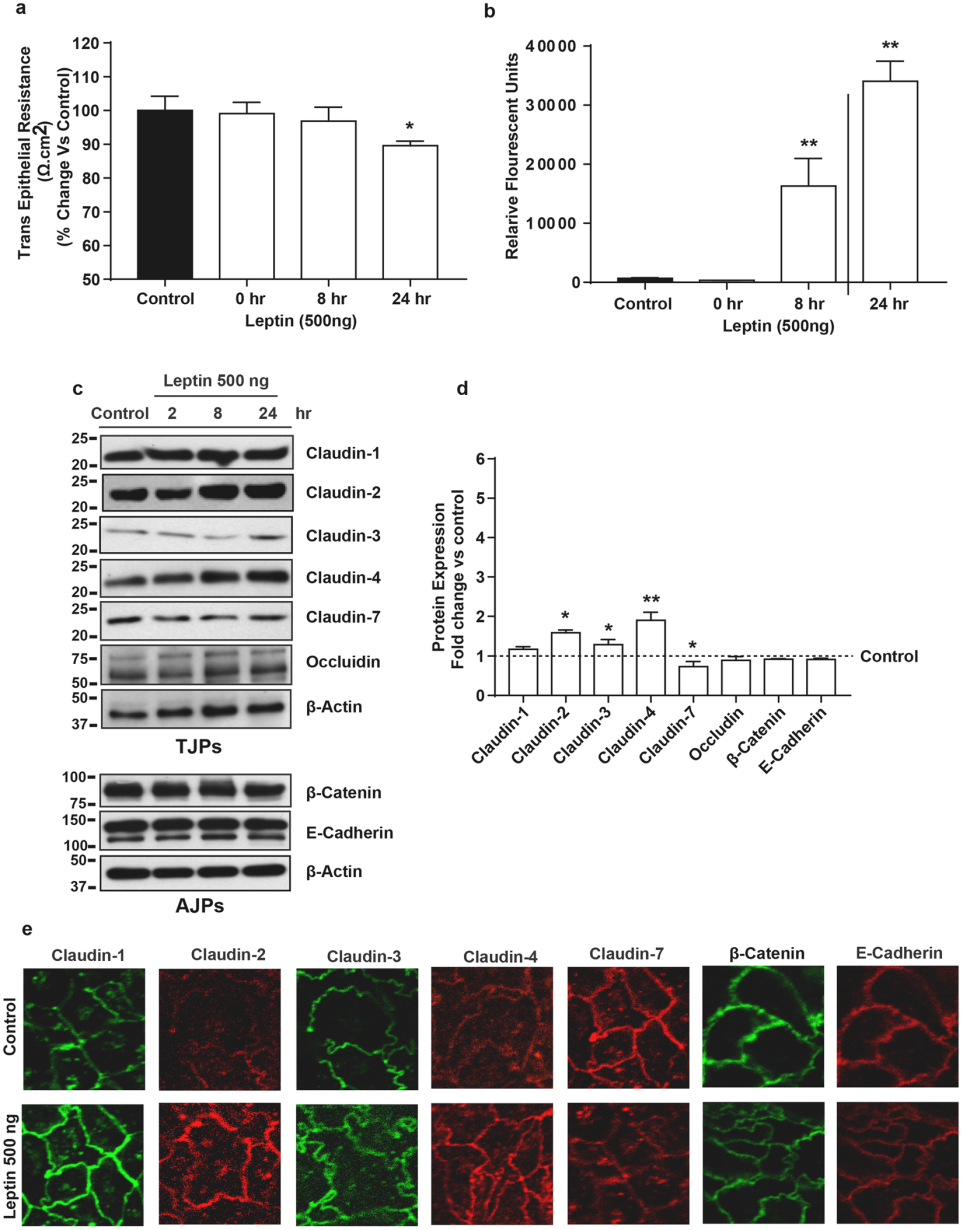
Leptin induced changes in intestinal epithelial barrier function are dependent on differential restructuring of tight junction than DCA-treatment. *Polarized monolayer of Caco-2 cells was subjected to Leptin (500 ng)-treatment (in complete culture medium) for different time-points*. (**a**) Effect of leptin-treatment upon trans-epithelial resistance across the cell monolayer; (**b**) Apico-basal paracellular permeability in control or leptin-treated cells as described in “materials and methods”. (**c**) Immunoblots analysis of total cell lysate from control and leptin treated cells by using antigen-specific antibodies; (**d**) Quantitative analysis of the antigen specific band intensity from immunoblot analysis; (**e**) Representative confocal immunoflurescent imaging to determine cellular localization of cell-adhesion proteins (TJPs and AJPs) in control and leptin-treated cells. Values are presented as mean ± SEM. **P ≤ 0.01 and *P ≤ 0.05 compared to control.

Similarly, after establishing differentiated polarized monolayer in transwell filter supports, Caco-2 cells were exposed to leptin (500 ng) for different time points (0, 8, and 24 hrs) to determine the effect upon TER and paracellular permeability. Similar to the DCA-treatment, leptin-treated cells also demonstrated a significant drop in TER (p < 0.05 versus control cells) but only after 24 hours’ post-treatment. The apico-basal permeability for FITC-Dextran dye also increased in these cells in a time dependent manner and was significantly higher at 8 and 24-hours post-treatment time-points (Fig. 6(a and b)). Interestingly, immunoblot demonstrated that leptin-induced claudin switching differs markedly compared to the DCA-induced tight junction restructuring. Specifically, the leptin-treatment significantly downregulated claudin-7 expression (p < 0.05) but increased claudin-2 (p < 0.05), claudin-3 (p < 0.05) and claudin-4 expressions significantly (p < 0.01) (Fig. 6(c and d)). However, similar to the DCA-treatment, leptin-treatment failed to induce significant changes in claudin-1, occludin, E-cadherin or beta-catenin expressions, compared to control cells (Fig. 6(c and d)). Immunofluorescent analysis supported the outcome from immunblot analysis (Fig. 6(e)). Taken together, our data suggested that differential tissue micro-environment, as suspected to be the case in obese versus lean individuals, can modulate tight junction’s claudin switching that may reflect defective barrier function and possibly altered epithelial homeostasis.

### In MDCK-II cells, Deoxycholic acid (DCA) and leptin-treatment inhibits claudin-2 expression

In the light of our *in vivo* data that obesity resulted in significant suppression of renal claudin-2 expression, contrasting the sharp increase in the intestinal epithelium, we examined if leptin and/or DCA exposure may have similar effects *in vitro*. Polarized monolayer of MDCK-II cells, widely used renal tubular epithelial cells, was subjected to leptin (250 ng/ml) or DCA (20 µm)-treatment, as above. Immunoblot analysis was done using samples collected 24-hours post-treatment. As demonstrated in Supplementary Fig. 1, both, leptin or DCA-treatment markedly suppressed claudin-2 expression, similar to the *in vivo* findings. Interestingly, expression of other claudin proteins remained largely unmodified in same samples (Supplementary Fig. 1). Taken together, these data supported tissue specific effect of obese microenvironment upon tight junction restructuring.

### TNFα/NF-kB/JUN MAP Kinase signaling regulates obesity-induced increase in claudin-2 expression in intestinal epithelium

**Figure 7:**
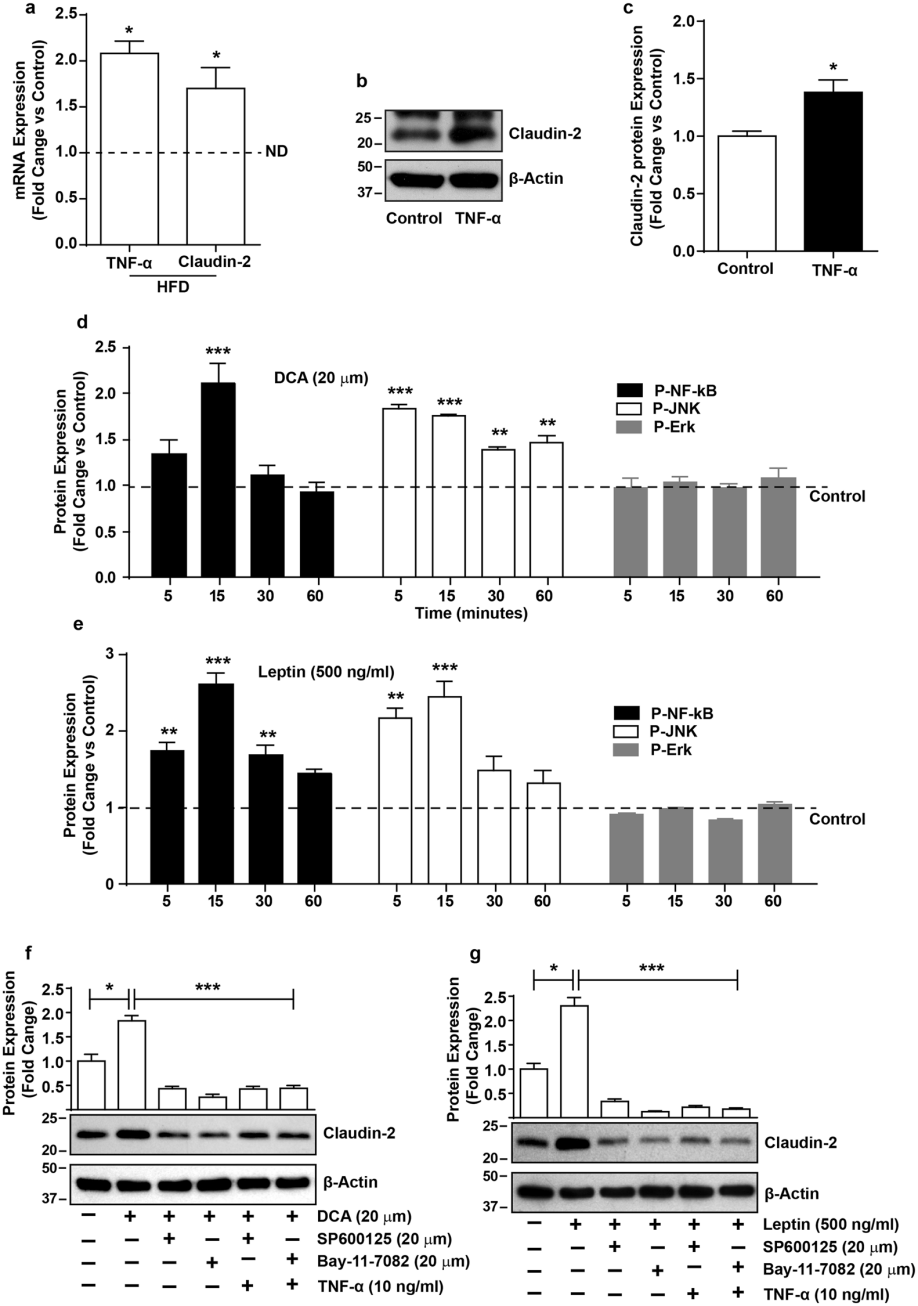
TNFα/NF-kB/JUN MAP-Kinase Signaling regulates obesity-induced increase in claudin-2 expression in intestinal epithelium. (**a**) qRT-PCR using total RNA isolated from control or HFD-fed mice colon; (**b**) Effect of TNF-α treatment upon claudin-2 expression in Caco-2 cells; (**c)** Quantitative analysis of the band intensity from immunoblot analysis; (**d** and **e**) Effect of leptin or DCA-treatments upon cellular signaling mechanisms and quantitative analysis of the antigen specific band intensity from immunoblot analysis; (**f** and **g**) Effect of inhibiting NF-kB or JUN MAP Kinase signaling using pathways specific inhibitors upon leptin or TNF-α induced increases in claudin-2 expressionand quantitative analysis of the antigen specific band intensity from immunoblot analysis; Values are presented as mean ± SEM. ***P ≤ 0.001, **P ≤ 0.01 and *P ≤ 0.05 compared to control.

Claudin-2 is one of the highly regulated claudin proteins and modified in response to diverse cellular signals^15, 30–32^. Therefore, we further examined specific signaling mechanisms underlying obesity-induced changes in claudin-2 expression. In particular, we examined potential role of the TNF-α signaling in this regulation as we found significant increase in TNF-α and claudin-2 mRNA expression in HFD-fed mice colon (Fig. 7(a)). Moreover, in Caco-2 cells subjected to TNF-α treatment (10 ng/ml), claudin-2 expression increased sharply (Fig. 7(b and c)). TNF-α promotes NF-kB-signaling and MAPK signaling^33, 34^. Therefore, we examined the possibility whether Leptin and/or DCA-induced claudin-2 upregulation in intestinal epithelium was dependent on similar signaling mechanisms. Caco-2 cells were subjected to Leptin or DCA-treatments and effects on diverse signaling pathways was determined. It was interesting that the leptin or DCA-treatment induced NF-kB and JUN MAP-Kinase signaling in time-dependent manner though the timings of the peak activation differed (Fig. 7(d and e)); Supplementary Fig. 2). In same samples, ERK1/2 MAP-Kinase activity remained largely unmodified (Fig. 7(d and e); Supplementary Fig. 2). To further determine, causal role of these signaling in obesity-induced claudin-2 expression, Caco-2 cells were subjected to leptin or DCA-treatments with or without pharmacological inhibitors for NF-kB (Bay-11-7082; 20 µm) and JUN MAP Kinase (SP600125; 20 µm) signaling pathways. Additional wells of these cells were subjected to TNF-α-treatment in the presence of same inhibitors. It was interesting that inhibiting either NF-kB or JUN MAP Kinase signaling inhibited the Leptin or DCA-induced claudin-2 upregulation (Fig. 7(f and g)).

### Biological processes enriched in claudin interacting proteins

**Figure 8:**
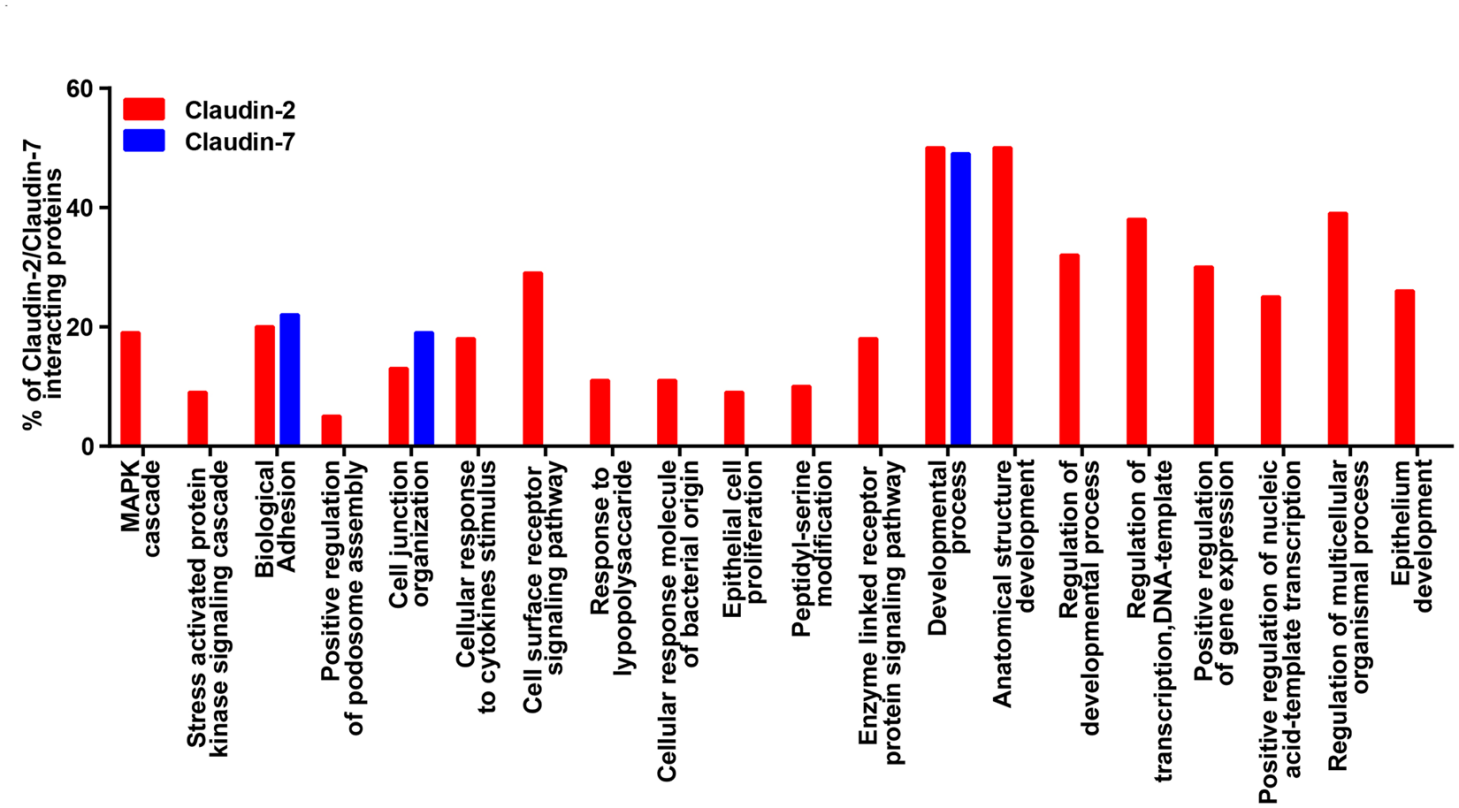
In silico analysis of claudin-2 and claudin-7 interaction in biological processes. Functional enrichment of proteins known to interact with claudin-2 and claudin-7: Many of the proteins that interact with claudin-2 encompass many biological processes including MAPK cascade and cell surface receptor signaling pathways, the proteins interacting with claudin-7 were enriched in fewer processes and were primarily involved in epithelial development.

Considering that the obesity and associated inflammation is foundation for pathological susceptibility, we performed an in-silico analysis to determine potential biological networking between claudin proteins and other signaling proteins. The STRING search operation focused especially on claudin-2 and claudin-7 interactions resulted in over 60 proteins that interacted with ‘claudin-2*’* and over 50 with claudin-7. Figure 8 shows a summary of PANTHER functional analysis of interacting proteins enriched in different biological processes^35^. Claudin-2 interacting proteins revealed enrichment of protein functions including MAPK cascade, kinase signaling cascade, response to cytokine stimulus and cell surface receptor signaling pathway. These proteins interacting with claudin-2 were often over expressed or activated in colorectal cancer (Supplementary Fig. 3(a), A ∩ E) but only Smad4 was the proteins that interacted with claudin-7 (Supplementary Fig. 3(b), A ∩ E). Similarly, the relation between claudin2 and calcium ion transport function was captured by A ∩ D (Supplementary Fig. 3(a) but claudin-7 interacting proteins did not show this function. Additionally, the relation between claudin-7 and sodium ion transport function was captured by A ∩ C. Supplementary Fig. 3(b), shows two proteins Wnk1 and Wnk2 with this function.

## Discussion

Obesity is a state of chronic subclinical systemic inflammation, which is postulated to predispose obese individuals to multiple metabolic disorders^30, 36^. Multiple plausible mechanisms have been proposed to explain the mechanistic undertakings for this causal association. However, consensus seems to exist for direct association between a hyper-permeable gut, characterizing obese individuals, and susceptibility to obesity-associated disorders. It is believed that a leaky gut helps enable the crossover of luminal microbial products, such as LPS, into the blood stream causing sub-optimal systemic inflammation^17, 37^. Also, the possibility that a leaky gut may help modulate gut microbiota by influencing the micro-environment cannot be ruled out. A critical role of gut microbiota in human health is now well recognized but the molecular undertakings of obesity-induced barrier deregulation remain ill understood. In the present study, using a comprehensive *in vivo* and *in vitro* analysis, we demonstrate organ and tissue-specific claudin switching as potential cause for obesity-associated tight junction restructuring and mucosal barrier deregulations. Our data that obesity-associated secrotomes induce variable changes in claudin expression, despite similar deregulation of intestinal epithelial cell barrier integrity, implicate greater roles of claudin proteins in obesity-associated pathobiology than the barrier deregulation. Overall, our findings highlight for the first time, in our knowledge, clinical significance of claudin expression, especially claudin-2, and tight junction restructuring in dealing with the obesity-associated epidemic.

These studies were performed using the validated approach of inducing obesity in mice by feeding high-calorie diet, the key detrimental factor in promoting obesity. Our findings that in HFD-mice, the trans-epithelial resistance was markedly decreased while crossover of luminally applied FITC-Dextran through rectum into the blood plasma was significantly higher, compared to the ND-mice, clearly supports deregulated tight junction properties and mucosal barrier properties. Our *in vitro* studies where polarized monolayer of cultured IEC was exposed to leptin, entrusted to regulate appetite and body weight and deregulated in obese individuals, clearly recapitulated the findings *in vivo*. Similar barrier deregulation in cultured IECs in response to DCA-exposure however accompanied by differential modulation of claudin protein expression implicates complex interaction between the tissue microenvironment, tight junction restructuring and barrier deregulation. As described, bile acid secretion is upregulated upon high-fat dietary intake^38^. Similar findings by other laboratories that obesity, leptin-treatment or DCA-treatment increases permeability across intestinal epithelium support our findings^38^.

The claudin family of transmembrane proteins is integral constituent of the tight junction and is expressed in tissue and in cell-specific manners^39^. Moreover, changes in claudin proteins, especially upregulation of claudin-2 protein, in association with pathological leakiness has been widely described^13, 15^. We have recently demonstrated that claudin-2 overexpression in intestinal epithelium in mice suffice to induce hyper-permeability^13^. However, in current study, obese mice gut epithelium presented diverse changes in tight- and adherent junction constituent proteins between small and large intestine. In this regard, studies have now highlighted discrete role of specific claudin proteins in regulating paracellular ion transport^40, 41^. The pathobiological significance of this function of claudin proteins in metabolic regulation is highlighted by recent findings that genetic inhibition of claudin-2 and -15 proteins, responsible for paracellular Na^+^ transport, resulted in malnourishment and premature death^42^. Here, inefficient functioning of the Na^+^-dependent transporters responsible for nutrient absorption from gut was found to be the underlying cause^42^. Notably, obese individuals have problem with effective digestion and nutrient absorption^43, 44^. Also, claudin-2 null mice demonstrate susceptibility to gallbladder stone^45^. In both cases, inefficient paracellular transport of Na^+^ due to the loss of claudin-2 and/or -15 appeared central to the pathological problems^42^. In our studies, we have found profound but tissue-specific alterations in claudin-2 and 15 proteins. Additionally, the in-silico analysis highlighted the key association between claudin-2 expression and Na^+^-homeostasis. A key role of deregulated Na^+^-homeostasis in obesity associated co-morbidities including hypertension is well documented^46^. Uniquely, claudin-2 protein also helps transport calcium and is a direct target for Vitamin-D receptor^47^. Significance of calcium homeostasis in health and disease including gastro-intestinal diseases is well established^48^. Overall, based on our data it is tempting to speculate that differential ion homeostasis, due to tissue specific claudin switching, may help modulate gut microenvironment and associated microbiota to promote barrier deregulation and disease susceptibility.

Here, it is notable that the changes in renal claudin-2 expression in obese mice contrasted the significant upregulation in gut epithelium *in vivo* and *in vitro*. Importantly, claudin-2 protein in the renal epithelium is expressed predominantly in the proximal tubular epithelium, the principal absorptive site for ions and solutes, including sodium and water in the kidney^11^. Accordingly, claudin-2 null mice demonstrate defective sodium handling and absorption^49^. Thus, the possibility that the reduced renal claudin-2 expression may impact blood Na^+^ -levels by waning proximal tubular reabsorption to aid to obesity-associated co-morbidities including hypertension cannot be ruled out. Additionally, the lack of renal claudin-2 expression was recently demonstrated to promote hypoxic environment and increased susceptibility to ischemic injury^26^. Similarly, renal claudin-7 deficiency is associated with renal salt wasting and chronic dehydration^50^. Interestingly, claudin-4 expressed predominantly in collecting ducts and serving as chloride permeability determinants was markedly upregulated in HFD diet mice kidney^51^. The increase in claudin-4 was however compensated by suppressed expression of the sister claudin protein, claudin-3, which share structural and functional properties with claudin-4. Taken together, renal claudin switch in HFD-mice (*versus* ND-mice) suggested profound alterations in renal ion homeostasis in HFD- *versus* ND-mice. However, elaborate studies with specific modulation of candidate proteins would be required for clear understanding of the role these proteins may play in regulating obesity associated metabolic disorder and co-morbidities.

Yet another consideration for the differential and tissue-specific changes in claudin expression could be their non tight-junctional functions. In this regard, loss of intestinal claudin-7 expression resulted in epithelial cell sloughing and spontaneous inflammation^52^. We have previously demonstrated a sharp decrease in claudin-7 expression in transformed intestinal epithelium and its causal association with carcinogenesis^53^. Similarly, a decrease in intestinal claudin-3 expression, as seen in our studies, has been associated with immature gut and barrier deregulations, and dedifferentiated gut epithelium^54, 55^. Claudin-1 expression is similarly associated with dedifferentiated gut epithelium depending upon its expression levels and cellular distribution^22, 56, 57^. On the other hand, decreased claudin-15 expression and an increase in claudin-2 expression in mice induce proliferation^13, 58^. Thus, taking into consideration the diverse however robust changes in specific claudin proteins, it is tempting to speculate that observed changes may reflect altered differentiated status of respective epithelia in obese mice. Of note, obese individuals are more susceptible to oncological transformations and growth including colon and renal cancers than healthy individuals^59, 60^.

An important question however remains regarding potential regulators of the tight junction remodeling in the gut and other organs in response to HFD-mice and obesity. We believe it is not the HFD that is responsible for the remodeling of the tight junction and barrier composition but it is the bodily responses to high caloric food intake. In this regard, excessive and prolong dietary fat increases levels of bile acids in intestinal lumen and leptin levels in blood circulation^61–64^. We hypothesize that the bile acids and leptin are the potential mediators of the observed claudin switch and barrier remodeling, possibly by modulating the local and/or systemic immune homeostasis. Our data from *in vitro* modelling supports such a postulation and implicates the role of TNF-α/NF-kB/JUN MAP-Kinase signaling in this regulation. A causal association between inflammatory signaling and barrier deregulation has previously been demonstrated and the role of the TNF-α and NF-kB signaling in regulating claudin-2 expression has been reported^30, 34, 65^. Our findings corroborate well with previous *in-vitro* reports showing that bile acids and fat emulsion treatment modulate barrier composition and function in IECs^38^. On the other hand, leptin secreted mostly from adipose tissue, regulate the energy homeostasis while leptin from gastric secretion inhibits intestinal sugar absorption by modulating sodium-glucose transporter 1 activity while luminal leptin enhances intestinal absorption of dietary proteins by stimulating peptide transport activity. Here, we demonstrated that apical leptin treatment significantly modulates tight junction composition by increasing claudin-2 and -3 expressions and decreasing claudin-7 expression, which might explain the decreased TER and increased paracellular permeability in polarized caco-2 cells. Our data further signifies the tissue-specificity of these effects as similar treatments of MDCK-II cells affect claudin expression differentially and inhibits claudin-2 expression.

Taken together, we here present, for the first time, a comprehensive analysis of the effects of high calorie and fat enriched diet upon gut permeability and tight junction restructuring. Our novel findings describe diverse and tissue specific changes in tight junction and adherent junction proteins in HFD-induced obesity versus ND-mice that is supportive of significant changes in barrier properties, ion homeostasis and epithelial homeostasis, which together may predispose obese individuals to differing pathological conditions.

## Methods

### Materials

High fat diet (HFD; 60% of total calories from fat) was obtained from the Bio Serv, New Jersey. The antibodies against claudin proteins Claudin-1 (Cat#717800), Claudin-2 (Cat#325600), Claudin-3 (Cat#341700), Claudin-4 (Cat#364800), Claudin-7 (Cat#374800) and Claudin-15 (Cat#389200) were purchased from Invitrogen Inc. (Carlsbad, CA), against E-cadherin (Cat#610181) and β-catenin (Cat#610153) from BD Biosciences (Franklin Lakes, NJ). The anti-β-actin antibody (Cat#A5441), deoxycolic acid (Cat#D2510) and leptin (Cat#L4146) were purchased from Sigma-Aldrich (St. Louis, MO). The human TNF-α was purchased from R&D Systems Inc. (Minneapolis, MN) while BAY-11-7082 (NFkB inhibitor) and SP600125 (JNK inhibitor) were obtained from Sigma Aldrich (St Louis, MO, USA).

The intestinal epithelial cells, Caco-2, were obtained from ATCC and cultured in DMEM-high glucose medium containing 10% FBS, 100 U/ml penicillin, and 100 µg/ml streptomycin. All cell culture reagents were from Invitrogen (San Francisco, CA, USA).

### High fat diet induced Obesity in mice

All experimental procedures were carried out in accordance with the Guidelines for the Care and Use of Laboratory Animals. All animal experiments in this study followed protocols approved by the Institutional Animal Care and Use Committee (IACUC) of University of Nebraska Medical Center, Omaha, Nebraska. C57BL/6 mice were purchased from the Jackson laboratory (Bar Harbor, ME) and then bred in our animal facility under specific pathogen–free conditions. Adult mice (8–10 weeks old) were used in this study. Mice had free access to normal chow (4% fat; control mice) or High fat chow (60% fat; experimental animals) except when fasted prior to the oral glucose tolerance test (OGTT). Food intake and body weight were monitored weekly and metabolic studies were performed as indicated below. Mice were sacrificed at 20 weeks’ post feeding of manipulated diet and small intestine, colon, adipose tissue, heart, liver, and kidney were harvested for further evaluations.

### Oral glucose tolerance test (OGTT)

The OGTT test was performed at the Mouse Metabolic Core at Vanderbilt University Medical Center (VUMC), Nashville, TN. In brief, fasted mice were weighed and fasting blood glucose level was measured immediately prior to the OGTT. A 50% glucose solution was given orally (2.5 g/kg) and blood glucose was measured at 15, 30, 60, and 120 min after glucose administration as described previously^38^.

### Determination of gut permeability

*In vivo* intestinal permeability assay to evaluate barrier deregulation was done using FITC-labeled dextran, MW 4000 (FD4 Sigma-Aldrich) as previously described^13^. Briefly, mice were orally administrated (gavage) FITC dextran-dextran with feeding needle (60 mg/100 g body weight of FITC-labeled dextran). Serum was collected retro-orbitally four hours later and fluorescence intensity was determined by using microplate fluorescence reader (excitation, 485 nm; emission, 528 nm; Bio-Tek).

### Western blot analysis

Immunoblot analysis was performed as previously described^57^. Signal was detected using an enhanced chemiluminescence detection kit (Amersham Biosciences). Equal protein loading was determined by re-probing with anti-β actin antibody after stripping the respective membrane.

### Trans-epithelial resistance and apico-basal permeability

For measurement of the trans-epithelial resistance (TER), caco-2 cell monolayers were grown on transwell filters (0.4 µm) and TER across the monolayer was assessed using an electrical resistance meter (Millipore, Bradford, MA). Cells cultured for at least three days, prior to experimental manipulations, to ensure polarized monolayer, were used. Measurements are presented as Ω⋅cm^2^ and expressed as percentage change compared to the baseline value. The paracellular flux for non-charged molecules was determined as described previously using FITC- dextran (4 kDa, Sigma-Aldrich Inc.)^15^. Data are presented as the total amount of FITC-dextran collected in the bottom chamber of transwell at indicated time points.

### Colonic trans-epithelial resistance measurement using the Ussing chamber system

Colonic epithelial sheets form HFD and ND-fed mice were harvested immediately after sacrificing and mounted in the dual channel Ussing Chamber system (Physiologic Instrument, San Diego, CA). The trans-epithelial resistance and conductance across the epithelium sheets were measured as previously described^13^.

### Immunohistochemistry

Immunofluorescent staining was done as described previously^56^. Briefly, cells were fixed with 95% ethanol for 20 min at 4 °C, then permeabilized with 0.2% Triton X-100 for 15 min. Immunofluorescence staining using tissues sections was performed using Tris-EDTA pH 9.0 buffer for antigen unmasking in relocking chamber (Biocare Medical, CA). Imaging for the immuno-labeled cells was performed using confocal microscope at the University of Nebraska Medical Center (UNMC) Imaging Core. All the images included in this manuscript were captured under identical microscope setting (for each antigen) and processed using the Adobe Photoshop (Adobe System, San Jose, CA) in identical manner.

### In silico analysis of tight junction proteins interaction

The complete mouse PPI network was obtained from STRING database and an induced sub-network was created using a gene list, which was obtained from multiple sources including STRING, GO and KEGG. Protein-protein interaction information was obtained for both claudin-2 and -7 protein, using STRING database^66^. Whereas all the genes annotated to have calcium/sodium ion transport functions were obtained from GO, the genes annotated with tight junction and colorectal cancer pathways were obtained from KEGG database. The proteins interacting with claudin-2 (or claudin-7) involved in multiple functions of interest were identified and labeled accordingly and visualized using Cytoscape (Supplementary Fig. 3a and b).

### Statistical analysis

Statistical analysis was done using Student’s t or one-way ANOVA in GraphPad Prism 6.0 (GraphPad Software, Inc.). A p-value < 0.05 was defined as statistically significant. All data presented are representative of at least three repeat experiments and presented as mean ± SEM.

## Electronic supplementary material

Supplementary Figures
