# Supplementary material for: Obesity-induces Organ and Tissue Specific Tight Junction Restructuring and Barrier Deregulation by Claudin Switching
Source: Sci Rep. 2017 Jul 11;7:5125. doi: 10.1038/s41598-017-04989-8 (PMC5505957; doi:10.1038/s41598-017-04989-8)
Supplement: Supplementary file 1 — Supplementary Figures [file 41598_2017_4989_MOESM1_ESM.pdf]

Obesity-induces Organ and Tissue Specific Tight Junction Restructuring and Barrier Deregulation by Claudin Switching

Rizwan Ahmad[1], Bilal Rah[1], Dundy Bastola[2], Punita Dhawan[1,3] and Amar B. Singh[1,3]

[1]Department of Biochemistry and Molecular Biology, University of Nebraska Medical Center, Omaha, NE 68198, [2]Department of Bioinformatics, University of Nebraska Omaha, and [3]VA Nebraska-Western Iowa Health Care System, Omaha, NE

Supplementary Figures Legends:

Supplementary Figure 1: Leptin or DCA-treatment suppresses claudin-2 expression in MDCK-II cells. *Polarized monolayer of renal tubular epithelial cells, MDCK-II cells, was subjected to DCA (20 µm) or Leptin (250 ng/ml)-treatment (in complete culture medium) for twenty-four hours.* a. Effect of DCA-treatment upon claudin expression using immunoblot analysis; **b.** Quantitative analysis of the antigen specific band intensity from immunoblot analysis; c. Effect of Leptin-treatment upon claudin expression using immunoblot analysis; **d.** Quantitative analysis of the antigen specific band intensity from immunoblot analysis; Values are presented as mean±SEM. ***P ≤ 0.001, **P ≤ 0.01 and *P ≤ 0.05 compared to control.

Supplementary Figure 2: DCA and Leptin treatment induces time dependent phosphorylation of NF-kB and JUN MAP Kinase in intestinal epithelium cells: Caco-2 cells was subjected time dependently to DCA and Leptin. Immunoblot analysis NF-kB, JUN MAP Kinase and ERK1/2 MAP Kinase during time dependent exposure of DCA and leptin.

Supplementary Figure-3: a. PPI sub-network induced by proteins interacting withclaudin-2 and proteins annotated with tight junction, Sodium/Calcium Ion Transport and Colorectal cancer related functions. Majority of proteins interacting with claudin-2 are annotated with tight junction function (A∩B). While these proteins are also found to be associate with colorectal cancer (A∩E), calcium ion transport (A∩D), non-was associated with sodium transport function (A∩C). Other overlapping functional relationships between two or more groups are also show. **; b.** PPI sub-network induced by proteins interacting with claudin-7 and proteins annotated with Tight Junction, Sodium/Calcium Ion Transport

and Colorectal cancer related functions. Majority of proteins interacting with claudin-7 are annotated with tight junction function (A∩B). While these proteins are also found to be associate with colorectal cancer (A∩E), calcium ion transport (A∩E), non-was associated with sodium transport function (A∩D). Other overlapping functional relationships between two or more groups are also show.

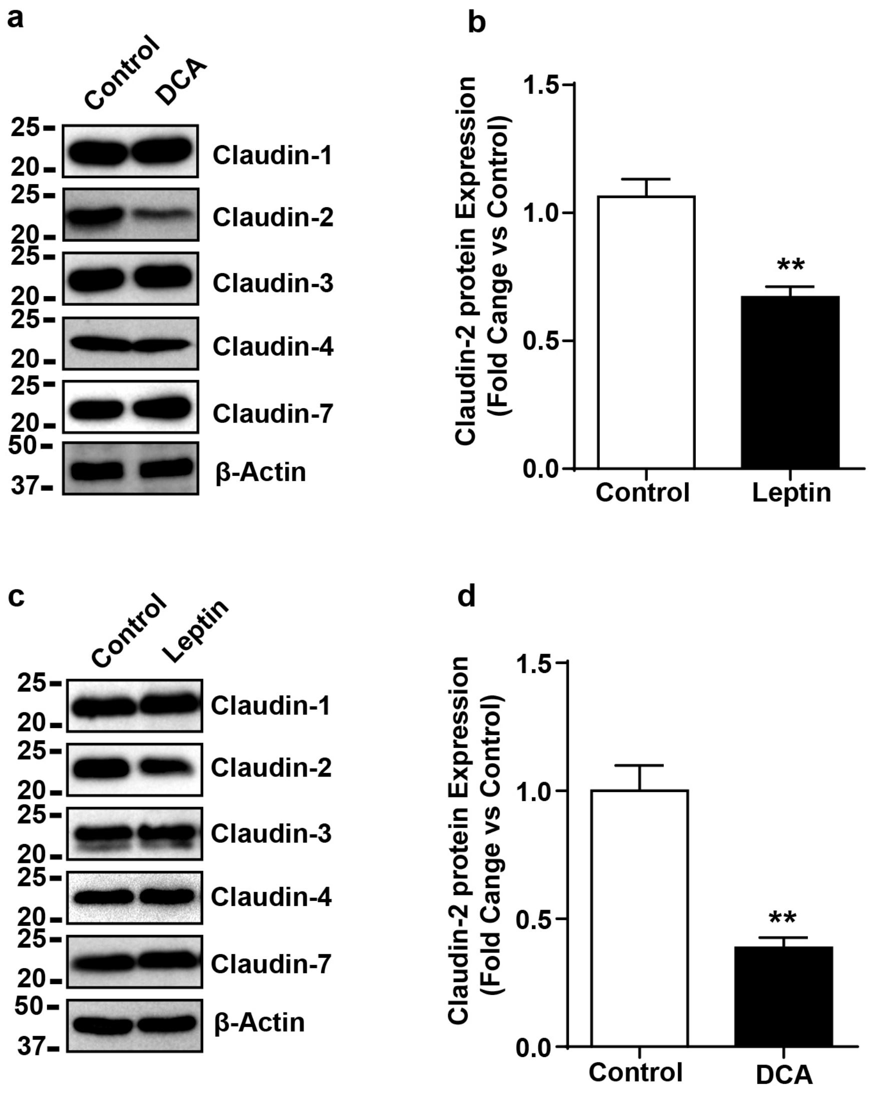

Supplementary Figure-1

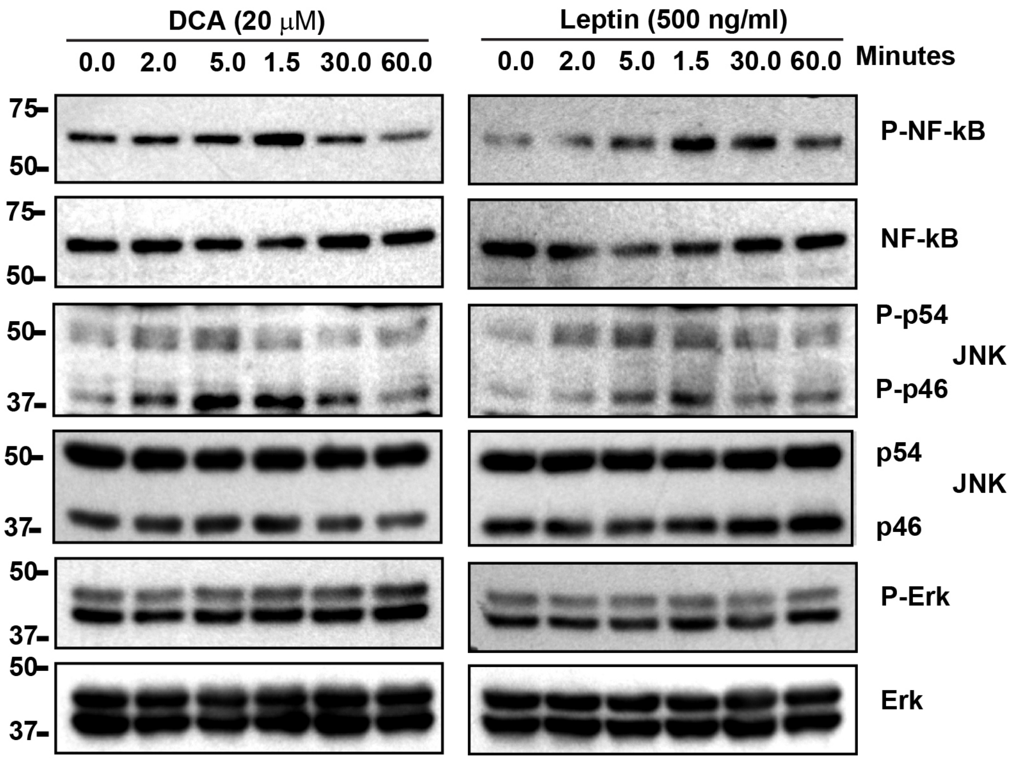

Supplementary Figure-2

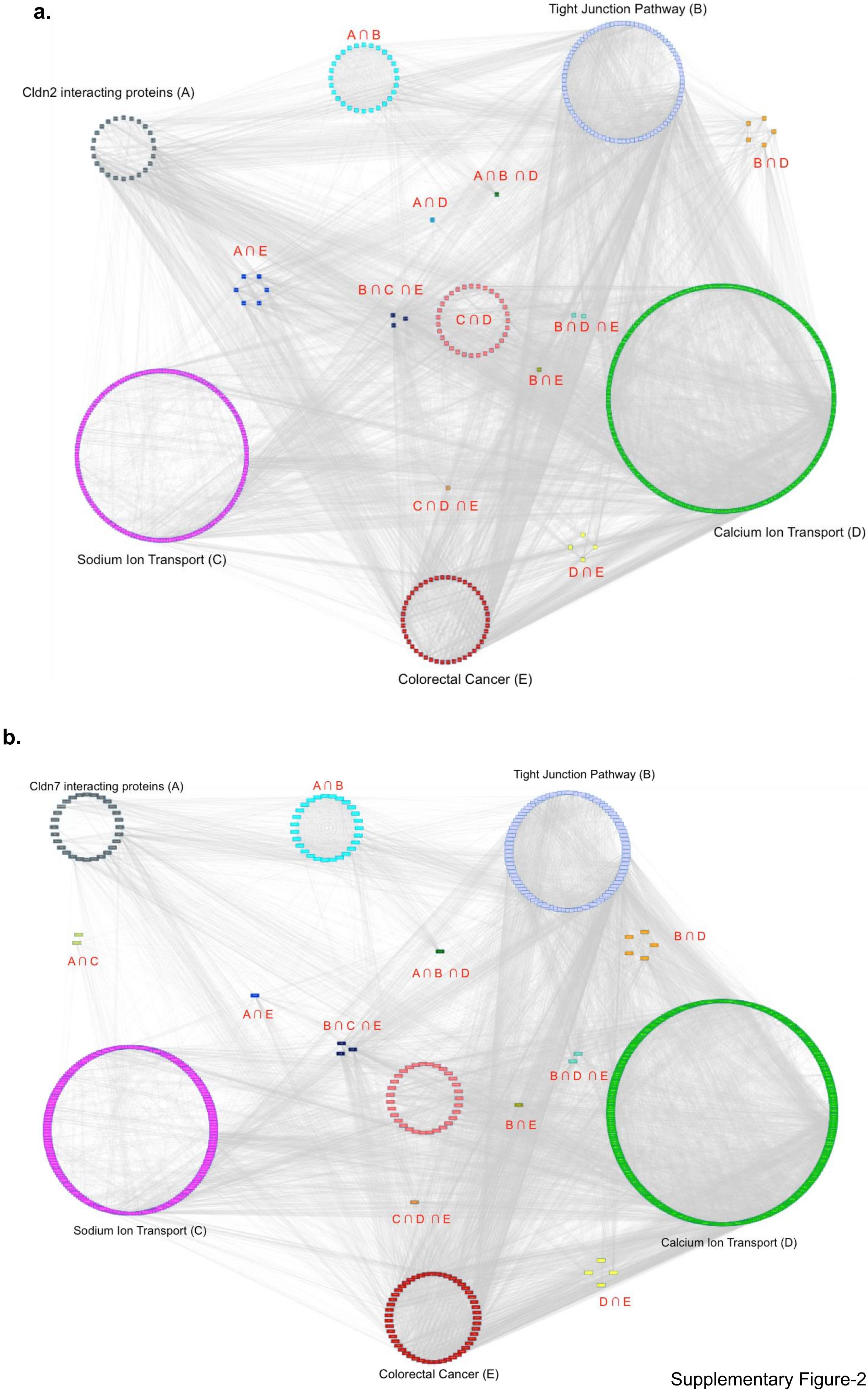

Supplementary Figure-2
